# Hypoxia-mediated activation of hypoxia-inducible factor-1α in head and neck squamous cell carcinoma: A review

**DOI:** 10.1097/MD.0000000000032533

**Published:** 2023-01-06

**Authors:** Lanxin Hu, Jinwei Hu, Yanlin Huang, Sihan Zheng, Ji Yin, Xiaohui Li, Daiying Li, Caifeng Lv, Sen Li, Wenjian Hu

**Affiliations:** a The Affiliated Traditional Chinese Medicine Hospital of Southwest Medical University, Luzhou, China; b Clinical Medicine, Nanchang University Queen Mary School, Nanchang, China.

**Keywords:** HIF-1α, HNSCC, Hypoxia, NF-κB, PI3K/AKT/mTOR, Ros

## Abstract

Since the 1950s, hypoxia has been recognized as a crucial characteristic of cancer cells and their microenvironment. Indeed, hypoxia promotes the growth, survival, and metastasis of cancer cells. In the early 1990s, we found that as many phenomena in hypoxia can occur through hypoxia-inducible factor-1α (HIF1α). HIF1α is known as an angiogenesis converter in hypoxia, which promotes tumorigenesis, development, immune escape, recurrence, etc; This page goes into great detail on how HIF1α is activated during hypoxia and how the 2 signaling channels interact. It specifically emphasizes the significance of reactive oxygen species, the function of the PI3K/the serine/threonine kinase Akt/mammalian target of rapamycin cascade, and outlines the similarities between the 2 important factors (reactive oxygen species and PI3K/the serine/threonine kinase Akt/mammalian target of rapamycin cascade), nuclear factor κB, for HIF1α Important implications, in an effort to offer fresh views for the treatment of head and neck squamous cell carcinoma and HIF1α research.

## 1. Introduction

Head and neck squamous cell carcinoma (HNSCC), which encompasses a number of squamous cell carcinomas that develop along the mucous membranes of the larynx, hypopharynx, oropharynx, and oral cavity and makes up more than 95% of all head and neck malignancies.^[1]^ With mortality rates of around 40% to 50%, HNSCC is the 6th largest prevalent malignancy worldwide.^[1,2]^ Alcohol and tobacco use, as well as high-risk human papillomaviruses, especially type human papillomaviruses-16, are the main risk factors for HNSCC.^[3]^ Because more than 80% of these tumors have an overexpression of the epidermal growth factor receptor (EGFR), the FDA approved Cetuximab, a monoclonal antibody against EGFR, for the treatment of HNSCC in 2006. However, It has only had modest success.^[2,4]^ Radiotherapy with or without chemotherapeutic drugs such as cisplatin is currently the norm for treating HNSCC.

Semenza and coworkers was initially discovered hypoxia-inducible factor-1 (HIF-1) in 1991 while conducting research on the erythropoietin (EPO) gene, a gene that produces red blood cells by producing the erythropoietin hormone.^[5]^ In this EPO gene’s 30-flanking region, cis-acting DNA sequences (50–RCGTG-30) were discovered. Hypoxia response elements are DNA sequences that have been identified to be required for the hypoxia-induced transcriptional activation of the EPO gene (HRE). Further research discovered that the binding of a specific protein to the HRE, which is brought on by hypoxia causes numerous regulatory genes to become transcriptionally active. Later, This protein was found to be HIF-1.^[6]^

Despite recent developments in survival rates, targeted therapy, and patient outcomes for HNSCC have not significantly increased in recent years, which, when combined with the disease’s increasing occurrence, increases the need for more potent treatment options.^[1,2,4]^Hypoxia or scarcity of chemical elements may be markers of solid tumors, and they adapt by activating HIF-1, a transcription problem that triggers the expression of quasi-links in nursing angiogenic molecules in an anoxic atmosphere. Redox state controls the expression of angiogenic molecules, including HIF-1, as a result of its activation. The tumor microenvironment can be affected in a number of ways through alterations in the redox atmosphere. Unfortunately, tumor hypoxia reduces tumor angiogenesis and radiation efficacy, increasing therapeutic resistance and cancer recurrence, as previously mentioned. The hypoxic environment is triggered by the HIF switch. HIF has an impact on a variety of physiological processes, including apoptosis, invasion/metastasis, immunity, cell proliferation, metabolism, and angiogenesis, through regulating over 100 genes.^[5,7]^ Translation of the HIF1α protein and mRNA are both upregulated when the signal pathway cascade of PI3K/The serine/threonine kinase Akt (AKT)/ mammalian target of rapamycin (mTOR) is activated.^[8]^ In actuality, hypoxia promotes growth, survival and the spread of cancerous cells. Hypoxia in the tumor is a common and significant stumbling block to effective cancer treatment.

To present, there is no FDA-approved medication to reverse tumor hypoxia, despite the fact that a variety of techniques have been used. A few inhibitors prevent the subunit dimerization of HIF-1 and/or HIF-2 mRNA or protein, or HIF interaction with co-activators; however, the majority are indirect inhibitors or have several actions. As a result, creating more targeted HIF inhibitors remains a significant problem. Unfortunately, due to safety concerns and low therapeutic efficacy, no medicines that directly inhibit the therapy of cancer patients, and HIFs have received authorization. In light of hypoxia-inducible factor-1α (HIF-1α’s) significant contributions in tumor microenvironment, in solid cancers like HNSCC, targeting and correcting hypoxia may be crucial to enhancing therapeutic response. The activation of HIF, including the redox-sensitive stages in HIF1α, the primary molecular actors involved, and their interaction with the reaction to unfolded proteins, as well as the influence of the PI3K/Akt pathway on HIF, and summarizes the common points of the 2, nuclear factor κB (NF-κB), for HIF1α Important implications, is covered in this paper. It offers a fresh approach to clinical research and treatment.

## 2. The relationship between HIF-1α and HIF-1 structure

**Figure 1.**
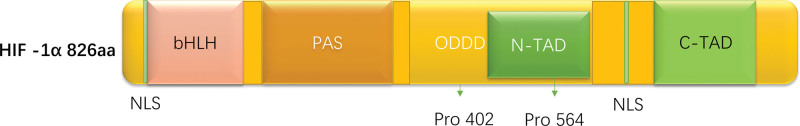
Functional domains of HIF-1α. ARNT = Aryl hydrocarbon receptor nuclear translocator, bHLH = basic-helix-loop-helix, C-TAD = C-terminal transactivation domain, HIF-1*α* = hypoxia-inducible factor-1α, N-TAD = N-terminal transactivation domain, ODDD = oxygen dependent degradation domain, PAS = Per/ARNT/Sim domain.

The heterodimeric transcription factor HIF-1 consists of 2 subunits: HIF-1α [or its analogs, hypoxia-inducible factor-2α (HIF-2α) and hypoxia-inducible factor-3α (HIF-3α)], and hypoxia-inducible factor-1β (HIF-1β).^[9]^ Low oxygen levels trigger the expression of the oxygen-sensitive subunit HIF-1α. HIF-1α, on the other hand, is expressed all of the time. Aryl hydrocarbon receptor’s heterodimeric partner, HIF-1β, has been discovered. Aryl hydrocarbon nuclear translocator (ARNT) is another name for HIF-1β (a heterodimeric partner of aryl hydrocarbon receptor [AhR]). HIF-1β links to AhR, allowing it to go to the nucleus more easily.^[9]^ These 2 components share structural similarities with 2 Drosophila nuclear proteins Per-ARNT-Sim (PAS) that include the basic-helix-loop-helix (bHLH) motif, making them members of the bHLH-PAS protein family.^[9]^ Identifiable domains set the bHLH proteins apart (b, HLH, PAS, and topologically assocaited domain) that it allows them to regulate their own transcription as well as the transcription of other family members. For HIF-1 and HIF-1 subunits to form heterodimers and bind to the HRE-DNA sequence on target genes, the bHLH-PAS motifs are necessary. Base domains’ ability to bind DNA was found to be necessary for the HRE to attach to the gene, whereas dimerization with other proteins takes place at the HLH motif. It was discovered that the only protein family member with a conserved domain is PAS (among HIF-1α, ARNT, AhR, and PAS), and is not found in any other proteins. The HIF-1α subunit has NH2-terminal N-terminal transactivation domain (N-TAD) and COOH-terminal transactivation domain transactivation domains topologically assocaited domain C-terminal transactivation domain (C-TAD). HIF-1α transcriptional activity is controlled by these 2 domains.^[10]^ Under hypoxia, CBP/p300 is 1 co-activator with which C-TAD interacts to control transcription of HIF-1α genes. HIF-1α is stabilized by N-TAD, which protects it against degradation.^[11]^ Furthermore, Since the oxygen-dependent degradation domain (ODDD) overlaps N-TAD in all HIF-subunit structures, they all differ from HIF-1β. This ODDD domain is crucial to controll the stability of O_2_ regulation.^[12]^ Three closely similar isoforms of the HIF-1 protein include HIF-1α, HIF-2α, and HIF-3α.^[10]^ Compared to HIF-3α, HIF-2α has a lot in common with HIF-1α in terms of the amino acid sequence (48% sequence similarity). This structural similarity may account for their ability to heterodimerize with HIF-1β and bind to HREs. HIF-1α and HIF-2α have varied tissue distribution patterns, which is interesting. While HIF-2α is only found in certain tissues, HIF-1α is expressed throughout the body.^[13]^ Before the identification of the inhibitory PAS (IPAS), a spliced variation of HIF-3α, HIF-3α was not well known. This domain is a dominant-negative regulator of HIF-1/DNA binding ability and lacks intrinsic transactivation activity in compared to the C-TAD of HIF-1α and HIF-2α.^[13]^ The activation of HIF1 Alpha in hypoxic conditions is the subject of the following thesis. The structure of the HIF-1α is shown in Figure 1.

## 3. The activation of HIF-1α

**Figure 2.**
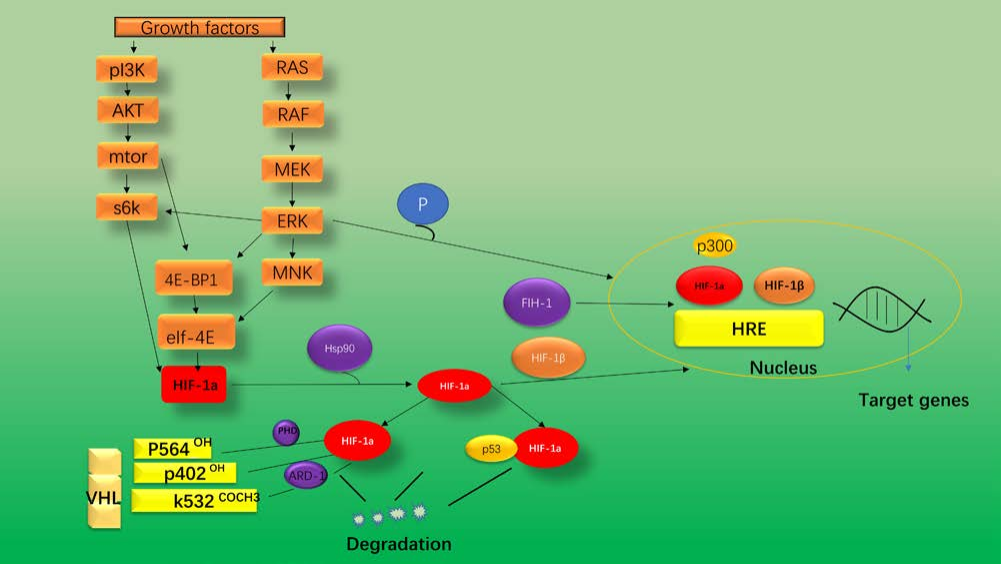
Regulation of HIF-1α pathway at different levels. (1) Growth factors related pathways; (2) pVHL related pathways; (3) FIH-1 pathway; (4) Mdm2-p53 mediated ubiquitination and proteasomal degradation pathway; (5) Hsp90. Ras/Raf/MEK: Rat sarcoma/rapidly accelerated fibrosarcoma/MAPK/ERK kinase. These pathways regulate HIF-1α activity by regulating HIF-1α synthesis, HIF-1α stability, or HIF1α transactivation. FIH = factor inhibiting hypoxia-inducible factor, HIF-1*α* = hypoxia-inducible factor-1α, MAPK = mitogen-activated protein kinase, pVHL = von Hippel Lindau protein.

Due to a series of signaling processes and other signaling molecules, HIF-1α is constitutively transcribed and generated regardless. Independently from O_2_ levels. In normoxic environments, HIF-1α rapidly degrades and has a brief half-life (approximately 5 minutes).^[14]^ Through post-translational modifications like hydroxylation, acetylation, ubiquitination, and phosphorylation processes, it has been demonstrated to regulate HIF-1 stability and transcriptional activity under hypoxic environments. As discussed below the above is mostly caused by the PI3K/Akt/MTOR pathway (Fig. 2). Following that, I’ll go into HIF regulation apart from the primary HIF regulatory pathways in hypoxia, including redox signaling in HIF-1α-dependent tumors. The regulatory aspects of various recent experimental studies were summarized, as well as the main regulatory role of PI3K/AKT/mTOR.

### 3.1. The role of redox signaling in hypoxia-induced HIF-1α activation

Reactive oxygen species (ROS) help to activate HIF-1α signaling. Late in the 1990s, It was found that the high levels of ROS produced by mitochondria during hypoxia can activate the HIF-1α signaling pathway.^[15]^ Numerous studies proposed prolyl hydroxylase (PHD) inactivation as a mechanism of ROS-mediated HIF-1α signaling activation.^[16,17]^which corroborated this finding. However, it is still uncertain how ROS mediates the stabilization of HIF-1α under hypoxia.^[14,18]^ Under normoxia, however, It is undeniable that ROS can keep the HIF-1α protein stable 14.ROS has been postulated as a method for oxidizing Fe2+, a co-factor of PHDs. Reactive oxygen species may affect the transcription and translation of HIF-1α through the ERK and PI3K/AKT/mTOR signaling pathways, as well as the synthesis of microRNA-210.^[14]^ Reactive oxygen species (ROS) are produced by mitochondria and maintain HIF stability. Complex III of the mitochondrial electron transport chain generates ROS.^[16,19]^ Another factor that could contribute to elevated ROS levels is the Circadian locomotor output cycle protein kaput.^[19]^ HIF-1α and NF-kB are both activated by ROS. ROS specifically impede the activity of PHD and factor inhibiting hypoxia-inducible factor in the cytoplasm.^[16,19,20]^ Some ROS-producing enzymes have a significant impact on ROS activation. The amounts of nitric oxide (NO) are required for HIF activation. This is significant since many malignancies are associated with a rise in inducible nitric oxide synthase (iNOS) expression and NO production, both of which make the patient’s prognosis worse.^[21]^ NO S-nitrosylates Cys533 on HIF-1, resulting in improved protein stability in normoxia. NO inhibits the activity of PHD by attaching to the iron atom at the catalytic core. Contrarily, NO can prevent HIF-1 from being activated and functioning in a hypoxic environment. When coupled with ROS, NO increases the amount of calcium ions in the cytoplasm, which causes calpain, a protease that eliminates HIF-1 without the help of the 26S proteasome, to be activated. Additionally, NO restores the function of the enzymes involved in chronic hypoxia’s HIF-hydroxylation through a process involving NO and ROS interaction.^[20,22]^

When HIF-1α binds to the von Hippel Lindau protein-E3 ubiquitin ligase complex, it becomes ubiquitinated and is subsequently targeted for degradation via the ubiquitin-proteasome pathway. In hypoxic conditions, prolyl hydroxylation occurs at a slower pace (i.e., when PHDs lack access to oxygen due to scarcity), reducing HIF-1a degradation.^[23]^ In the nucleus, stabilized HIF-1a accumulates, which induces the expression of a large number of proangiogenic factors, including vascular endothelial growth factor (VEGF), VEGF receptors (VEGFR), Angiopoietin (ANG-1 and ANG-2) receptor TIE-2, platelet derived growth factor-B, plasminogen activator mitogen 1, and certain matrix metalloproteinase family members (MMP-2 and MMP-9).^[24,25]^ Despite the fact that they can both heterodimerize with HIF-1β and bind to genes that are induced by hypoxia and have the hypoxia response elements motif, HIF-1α and HIF-2α have different transcriptional targets. Two glycolytic enzymes, lactate dehydrogenase-A and carbonic anhydrase, are stimulated by HIF-1α. On the other hand, HIF-2α has a higher impact on the genes involved in iron metabolism and the EPO gene, but both HIF-1α and HIF-2α regulate VEGF and GLUT-1.^[9]^ VEGFA, an endothelial mitogen, is the most notable of these HIF-1/2 targets because it is considered to be the primary controller of tumor angiogenesis. HIF-1α hydroxylation is catalyzed by prolyl hydroxylases, which introduce 1 atom of molecular oxygen into the cosubstrate a-ketoglutarate, which is then converted to succinate, and the other atom into the cosubstrate a-ketoglutarate.^[23,26]^ PHD activity is inhibited by dimethyloxalylglycine, a competitive antagonist of a-ketoglutarate, which promotes the transcription of angiogenic factors reliant on HIF-1. Similarly, iron chelators and cobalt chloride, which increase HIF-1 activity and inhibit PHD by removing Fe (II) from the catalytic core.^[23,27]^ Redox active chemicals that have an impact on PHDs indirectly control the activity of hypoxia-inducible factor-1. A good illustration of this kind of chemical is ascorbate. It transforms ferric iron Fe (III) complexes that are insoluble in PHDs into stable ferrous Fe (II) chelates that are soluble. This demonstrates ascorbate’s function in chelating and reducing PHD-bound Fe (III) during hydroxylation, which maintains the enzyme cycle. Ascorbate functions in oncogenic activated cells as a cofactor of PHDs, reducing hypoxia- or iron chelators and cobalt chloride-induced HIF-1a activity in culture, indicating that it might increase PHDs’ HIF-1hydroxylase activity.^[28,29]^ Ascorbate supplementation decreased tumor metastasis and necrosis in ascorbate-deficient Gulo/ mice alongside the MMP-9 and VEGF production, which are HIF-1 targets, It suggests that ascorbate may slow tumor growth by preventing HIF-1.^[6]^Under normal circumstances, cellular antioxidant mechanisms keep ROS below spectrum of physiological conditions necessary for effective cell signaling. ROS levels can rise quickly due to increased ROS production or a lack of antioxidant defense, which can influence HIF-1 signaling (as demonstrated in a variety of tumor types).

In fact, ROS can facilitate iron oxidation, which stabilizes HIF-1α and inactivates PHD. Other redox posttranslational changes have been shown to control HIF-1α turnover in addition to hydroxylation. Numerous studies have discovered that HIF-1a is targeted by nitric oxide (NO) via S-nitrosylation at Cys533, a crucial cysteine within HIF-1a’s oxygen-dependent degradation domain. Even under normoxia, HIF-1a S-nitrosylation has been shown to stabilize the protein and block its connection with VHL, simulating pseudo-hypoxic circumstances.^[24]^ However, NO’s activation of HIF-1 signaling is not always straightforward. Indeed, hypoxia has been shown to cause HIF-1a stabilization indirectly through the nitrosylation of Fe (II) situated in the catalytic region of PHDs, which inhibits PHDs hydroxylase activity.^[30]^HIF-1 has long been thought to be a promising therapeutic target for cancer treatment because of its well-known function in tumor angiogenesis. Both direct HIF-1 inhibitors that affect HIF-1 expression or activity and indirect HIF-1 inhibitors that act on additional molecules in associated pathways have been discovered. HIF is inhibited by the first class of inhibitors through a variety of mechanisms, including mRNA expression, protein synthesis, dimerization, DNA binding, and transcriptional activity. However, only a handful of them are being studied in clinical settings because of their pleiotropic (side) effects. The mitochondrial complex I inhibitor BAY 87 to 2243 is one of these and prevents HIF-1α stability, HIF-1 translation is inhibited by PX-478 and HIF-1α translation is inhibited by KCN-1 and its transcriptional coactivator p300 association.^[31–33]^

### 3.2. ROS-generating enzymes: modulators of the redox signaling

Redox signaling modulators are proteins that regulate redox signaling. Many cellular enzymes, including endoplasmic oxidoreduction1, nicotinamide adenine dinucleotide dinucleotide, oxidases, the mitochondrial electron transport chain complexes I and III, and NOS, have been shown to have an immediate impact on angiogenic mediator levels and activity.^[2,21,22,34–38]^ The regulation of some ROS-generating enzymes, their function in angiogenesis, and how their activity is related to HIF-1 will all be discussed in this section.

### 3.3. The important pathway regulation of HIF-1α

Oncogenic stimulation of the PI3K/AKT/MTOR pathways has a significant role in modulating HIF-1α protein levels. Under hypoxic conditions, HIF-1α protein is known to accumulate in cells as a result of growth factors, cytokines, and other signaling molecules.

#### 3.3.1. Triggered of ERK through PI3K/AKT.

Signaling pathways for growth factors PI3K protein can be upregulated by activating it.^[8,39,40]^ Through rapamycin’s downstream component, mammalian target of and target protein kinase B Akt, PI3K regulates protein synthesis mTOR. By phosphorylating the eukaryotic translation initiation factor 4E binding protein, mTOR prevents cap-dependent mRNA translation eukaryotic translation initiation factor4e-binding protein1, causing these 2 components’ integrity to be compromised, which increases the translation of the HIF-1 protein.^[40]^ Furthermore, protein translation is triggered by mTOR-mediated phosphorylation of p70 S6 kinase, which increases the phosphorylation of its substrate, ribosomal protein S6.^[40]^ Tumor suppressor protein phosphatase and tensin homolog (PTEN) prevents PI3K products from being phosphorylated. Certain growth hormones cause rat sarcoma (RAS) to become active, which encourages the RAS/RAF/MEK/ERK kinase cascade.^[39]^ ERK (MNK) phosphorylate eukaryotic translation initiation factor4e-binding protein1, S6 kinase, eukaryotic translation initiation factor 4E,and MAP kinase interacting kinase.^[17]^ These signaling events increase the rate at which mRNA is translated into the HIF-1 protein as their overall effect. It’s interesting to note that ERK controls both HIF-1 synthesis and transcriptional activation. The co-activator CBP/p300 is phosphorylated by ERK, which improves the formation of the HIF-1/p300 complex and subsequently increases transcriptional activity.^[41]^ Hypoxia, both chronic and intermittent, is a cancer-causing factor.

#### 3.3.2. Triggered of mitogen-activated protein kinase (MAPK) through PI3K/AKT.

Hypoxia, AP-1 Kinases, and MAPK The physiological response to low oxygen levels is greatly influenced by MAPK cascades, which are triggered during hypoxia. Reactive oxygen species or the presence of calcium ions are required for the activation of MAPK pathways in hypoxia ROS.^[42]^ When there is hypoxia, *L*-type voltage-gated Ca2 + channels open, which results in a surge in calcium ions.^[42]^ oxygen species ROS or calcium ions activate the p38 MAPK and the c-Jun N-terminal kinase (JNK) MAPK.^[42,43]^ The levels of c-Fos and JunD MAPK cascade activity decrease as c-Jun and JunB are activated.on the other hand, does not just activate HIFs; HIF-1 could potentially influence MAPK cascade activation in the other manner.^[44]^ Under chronic hypoxia, Increased c-Jun expression in mouse embryonic fibroblasts is dependent on HIF-1, this is comparable to the ERK MAPK cascade being activated.^[25,45]^ C-Jun and JunB, which are part of the activating protein-1 (AP-1) complex, are crucial in the regulation of a number of genes under hypoxia. MAPK pathways can alter HIF1α activation and transcriptional activity. The ERK MAPK, p38 MAPK and JNK MAPK pathways are all activated by hypoxia.^[44,46,47]^HIF-1α is phosphorylated on Ser641 and Ser643 by ERK MAPK, p38 MAPK, JNK MAPK ERK. HIF-1’s association with the p300 coactivator and build up in the nucleus of the cell are both influenced by this post-translational change.^[46]^ Additionally, p38 MAPK phosphorylates HIF-1α, which improves this HIF-1 subunit’s stability.^[46]^ The 7 in absentia homolog 2 is phosphorylated by the p38 MAPK protein at the sites Thr24 and Ser29, which is another way affecting HIF-1α activation. PHD3, the enzyme that hydroxylates and degrades HIF-1α, then breaks down after this. HIF-1α is not degraded as much when p38 MAPK is activated. Dual specificity protein phosphatase (DUSP-1) and mitogen-activated protein kinase phosphatase 1 expression are upregulated in response to hypoxia.^[48]^ generated by neuronal nitric oxide synthase, nitric oxide (NO) (nNOS) and hypoxia are required for the enhanced expression of DUSP-1 in neurons. Contrary to how NO deactivates DUSP-1, protein kinase C increases DUSP-1 expression in fibroblasts during persistent hypoxia.^[49,50]^ Deferoxamine and cobalt chloride may also prevent histone demethylase activity, resulting in altered gene expression.^[49,50]^ HIF-1α also reduces DUSP-2 expression in chronic hypoxia, Enzymes called DUSP-1 and DUSP-2 dephosphorylate ERK and p38 MAPK to render them inactive.^[51]^ In chronic hypoxia, increase of DUSP-1/mitogen-activated protein kinase phosphatase 1 expression is a protective mechanism against excessive HIF1α activation by ERK MAPKs. In hypoxia, MAPK cascades improve HIF1α stability and transcriptional activity. Similar pathways are present in tumor cells under normoxia, where MAPK cascades are activated,^[52]^ particularly in response to different growth stimuli.^[53]^ MAPK kinases are activated by hypoxia, and NF-KB is then also activated. However, the MAPK cascade that causes this varies from model to model. In the case of HNSCC, more research is required. In the case of HNSCC, more research is required.

Although it is also regulated at the transcriptional and translational levels, HIF-1 is predominantly regulated at the protein level. When the PI3K/AKT/mTOR signaling cascade is active, HIF1α protein translation and mRNA transcription are both increased.^[54]^ Multiple growth factors, oncogene activation, and tumor suppressor gene alterations (such as PTEN) could all stimulate HIF1α signaling via the PI3K/AKT/mTOR pathway.^[54]^ According to reports, Glycogen Synthase Kinase-3 phosphorylates the HIF1α protein, causing VHL-independent HIF1α protein degradation.^[55]^ We further show that lower levels of PTEN, as a result of genetic silencing and pharmacological suppression of PTEN, cause HIF-1a to accumulate in tumor cells.^[54]^ The PI3K/AKT signal, in particular, has been shown to inactivate glycogen synthase kinase-3, resulting de HIF1α protein stabilization.^[56]^ The RAS/RAF/MEK/ERK cascade could be activated by a variety of growth factors and oncogenic events, speeding up the production of the HIF1α protein.^[57]^ By phosphorylating CBP/p300, a crucial co-factor for HIF1 transcriptional activity, ERK activation also increases HIF1 transcriptional activity.^[45,57]^

## 4. Important link of ROS and PI3K/AKT/mTOR pathway for HIF1α

**Figure 3.**
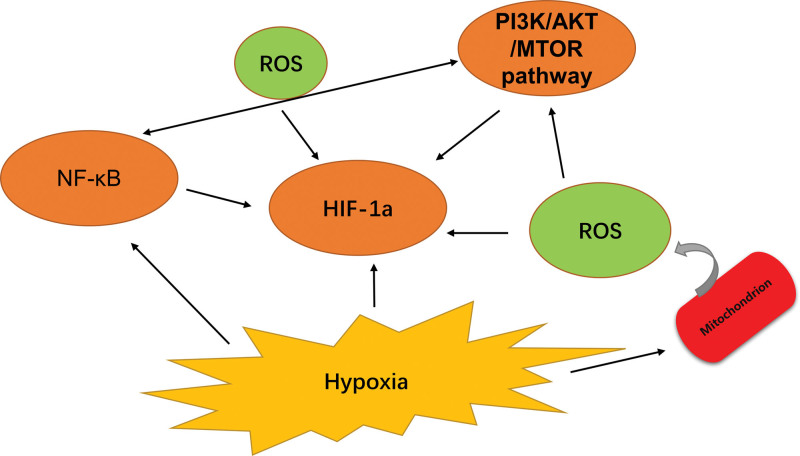
The parts influence each other, NF-κB is an important link between ROS and PI3K/AKT/mTOR. AKT = the serine/threonine kinase Akt. mTOR = mammalian target of rapamycin, NF-κB = nuclear factor κB, ROS = reactive oxygen species.

The ROS state regulates many cellular proteins. This process promotes the activity of growth factors, cytokines, and hormones by activating a number of proteins, including kinases, may activate downstream signaling pathways including phosphoinositide 3-kinase (PI3K) and mitogen-activated protein kinase (MAPK).^[41,58]^ As a result, ROS directly oxidize phosphatase. Other pathways must be activated for the greatest HIF activity. Particularly during the first few hours of persistent hypoxia, NF-κB is activated.^[59]^ Because the HIF1α gene promoter has an NF-κB binding site, HIF-1 mRNA expression is boosted.^[60,61]^ In order to boost HIF-1a mRNA expression, P50 NF-κB and P65/RelA NF-κB were activated, but not c-REL NF-κB.^[62,63]^ Additionally, various elements of the NF-κB activation pathway are impacted by chronic hypoxia.^[64]^ In hypoxia, the MAPK cascade is also triggered, which causes NF-κB to become active. In addition to these mechanisms, NF-κB occurs in hypoxia through the phosphatidylinositol 3-kinase (PI3K) →AKT pathway.^[65]^ This transcription factor is activated by Akt/PKB kinase’s phosphorylation of P65/RelA NF-κB, however this mechanism is unrelated to IκBα degradation. Moreover, TNF-α (tumor necrosis factor alpha) activates the NF-κB pathway when relevant components translocate into the nucleus, causing alterations to occur at the level of HIF1α^[66,67]^ (Fig. 3). We can see that both ROS and PI3K/AKT/MTOR pathways have NF-κB effects in activating HIF 1α. The effect of NF-κB on HIF 1α is self-evident.

## 5. Concluding

HIF1α signaling activation can be mediated by a number of hypoxia-independent pathways, according to growing evidence.^[57]^ Numerous important genes involved in tumor development, metastasis, and therapy resistance are controlled by HIF1α.^[41,68]^ As already mentioned, in hypoxic conditions, a number of variables (ROS, PI3K, and NF-κB) can stabilize HIF1α and benefit cancer cells by promoting cell motility, invasion, immunological escape, and radio resistance.^[18]^ We also described the NF-κB (the important link of ROS and PI3K/AKT/mTOR cascade) for HIF1α implications, in an effort to offer fresh views for the treatment of head and neck squamous cell carcinoma (HNSCC) and HIF1α research. It is beneficial to provide fresh evidence for HIF inhibitor or combination inhibitor research and promote clinical treatment by examining the elements that activate HIF1α and discovering common ground among them. Thus, it offers a fresh outlook on the creation of combination inhibitors.

## Author contributions

**Conceptualization:** Lanxin Hu, Wenjian Hu.

**Methodology:** Ji Yin.

**Resources:** Xiaohui Li, Sen Li.

**Supervision:** Jinwei Hu, Caifeng Lv, Sen Li, Wenjian Hu.

**Validation:** Lanxin Hu, Jinwei Hu.

**Visualization:** Lanxin Hu, Jinwei Hu, Ji Yin, Xiaohui Li, Daiying Li.

**Writing – original draft:** Lanxin Hu, Yanlin Huang.

**Writing – review & editing:** Lanxin Hu, Sihan Zheng.

## References

[1] Leemans CR Snijders PJF Brakenhoff RH. The molecular landscape of head and neck cancer. Nat Rev Cancer. 2018;18:269–82. 29497144 10.1038/nrc.2018.11

[2] Alsahafi E Begg K Amelio I. Clinical update on head and neck cancer: molecular biology and ongoing challenges. Cell Death Dis. 2019;10:540. 31308358 10.1038/s41419-019-1769-9 PMC6629629

[3] Economopoulou P de Bree R Kotsantis I. Diagnostic tumor markers in head and neck squamous cell Carcinoma (HNSCC) in the clinical setting. Front Oncol. 2019;9:827. 31555588 10.3389/fonc.2019.00827 PMC6727245

[4] Solomon B Young RJ Rischin D. Head and neck squamous cell carcinoma: genomics and emerging biomarkers for immunomodulatory cancer treatments. Semin Cancer Biol. 2018;52:228–40. 29355614 10.1016/j.semcancer.2018.01.008

[5] Semenza GL Nejfelt MK Chi SM. Hypoxia-inducible nuclear factors bind to an enhancer element located 3’ to the human erythropoietin gene. Proc Natl Acad Sci USA. 1991;88:5680–4. 2062846 10.1073/pnas.88.13.5680 PMC51941

[6] Kallio PJ Pongratz I Gradin K. Activation of hypoxia-inducible factor 1: posttranscriptional regulation and conformational change by recruitment of the Arnt transcription factor. Proc Natl Acad Sci USA. 1997;94:5667–72. 9159130 10.1073/pnas.94.11.5667 PMC20836

[7] Fasano M Della Corte CM Viscardi G. Head and neck cancer: the role of anti-EGFR agents in the era of immunotherapy. Ther Adv Med Oncol. 2021;13:175883592094941. 10.1177/1758835920949418 PMC7953226 33767760

[8] D’Angelo G Duplan E Boyer N. Hypoxia up-regulates prolyl hydroxylase activity. J Biol Chem. 2003;278:38183–7. 12876291 10.1074/jbc.M302244200

[9] Wang GL Jiang BH Rue EA. Hypoxia-inducible factor 1 is a basic-helix-loop-helix-PAS heterodimer regulated by cellular 02 tension. Proc Natl Acad Sci USA. 1995;92:5510–4. 7539918 10.1073/pnas.92.12.5510 PMC41725

[10] Li H Ko HP Whitlock JP. Induction of phosphoglycerate kinase 1 gene expression by hypoxia. J Biol Chem. 1996;271:21262–7. 8702901 10.1074/jbc.271.35.21262

[11] Infantino V Santarsiero A Convertini P. Cancer cell metabolism in hypoxia: role of HIF-1 as key regulator and therapeutic target. IJMS. 2021;22:5703. 34071836 10.3390/ijms22115703 PMC8199012

[12] Bruick RK McKnight SL. A conserved family of prolyl-4-hydroxylases that modify HIF. Science. 2001;294:1337–40. 11598268 10.1126/science.1066373

[13] Ema M Taya S Yokotani N. A novel bHLH-PAS factor with close sequence similarity to hypoxia-inducible factor 1regulates the VEGF expression and is potentially involved in lung and vascular development. Proc Natl Acad Sci USA. 1997;94:4273–8 9113979 10.1073/pnas.94.9.4273 PMC20712

[14] Movafagh S Crook S Vo K. Regulation of hypoxia-inducible factor-1a by reactive oxygen species: new developments in an old debate: regulation of hypoxia-inducible factor-1a. J Cell Biochem. 2015;116:696–703. 25546605 10.1002/jcb.25074

[15] Chandel NS Maltepe E Goldwasser E. Mitochondrial reactive oxygen species trigger hypoxia-induced transcription. Proc Natl Acad Sci USA. 1998;95:11715–20. 9751731 10.1073/pnas.95.20.11715 PMC21706

[16] Guzy RD Hoyos B Robin E. Mitochondrial complex III is required for hypoxia-induced ROS production and cellular oxygen sensing. Cell Metab. 2005;1:401–8. 16054089 10.1016/j.cmet.2005.05.001

[17] Koivunen P Hirsilä M Günzler V. Catalytic properties of the asparaginyl hydroxylase (FIH) in the oxygen sensing pathway are distinct from those of its prolyl 4-hydroxylases. J Biol Chem. 2004;279:9899–904. 14701857 10.1074/jbc.M312254200

[18] Rankin EB Giaccia AJ. Hypoxic control of metastasis. Science. 2016;352:175–80. 27124451 10.1126/science.aaf4405 PMC4898055

[19] Tang X Guo D Lin C. hCLOCK causes rho-kinase-mediated endothelial dysfunction and NF- κ B-mediated inflammatory responses. Oxid Med Cell Longevity. 2015;2015:1–9. 10.1155/2015/671839 PMC4637096 26583060

[20] Köhl R Zhou J Brüne B. Reactive oxygen species attenuate nitric-oxide-mediated hypoxia-inducible factor-1α stabilization. Free Radic Biol Med. 2006;40:1430–42. 16631533 10.1016/j.freeradbiomed.2005.12.012

[21] Radi R. Oxygen radicals, nitric oxide, and peroxynitrite: redox pathways in molecular medicine. Proc Natl Acad Sci USA. 2018;115:5839–48. 29802228 10.1073/pnas.1804932115 PMC6003358

[22] Calabrese C Iommarini L Kurelac I. Respiratory complex I is essential to induce a Warburg profile in mitochondria-defective tumor cells. Cancer Metab. 2013;1:11. 24280190 10.1186/2049-3002-1-11 PMC4178211

[23] Jaakkola P Mole DR Tian YM. Targeting of HIF-α to the von Hippel-Lindau Ubiquitylation Complex by O_2_ -regulated prolyl hydroxylation. Science. 2001;292:468–72. 11292861 10.1126/science.1059796

[24] Catrina SB Zheng X. Hypoxia and hypoxia-inducible factors in diabetes and its complications. Diabetologia. 2021;64:709–16. 33496820 10.1007/s00125-021-05380-z PMC7940280

[25] Kierans SJ Taylor CT. Regulation of glycolysis by the hypoxia-inducible factor (HIF): implications for cellular physiology. J Physiol. 2021;599:23–37. 33006160 10.1113/JP280572

[26] Kaelin WG Ratcliffe PJ. Oxygen sensing by metazoans: the central role of the HIF hydroxylase pathway. Mol Cell. 2008;30:393–402. 18498744 10.1016/j.molcel.2008.04.009

[27] Schödel J Ratcliffe PJ. Mechanisms of hypoxia signalling: new implications for nephrology. Nat Rev Nephrol. 2019;15:641–59. 31488900 10.1038/s41581-019-0182-z

[28] Reang J Sharma PC Thakur VK. Understanding the therapeutic potential of ascorbic acid in the battle to overcome cancer. Biomolecules. 2021;11:1130. 34439796 10.3390/biom11081130 PMC8392841

[29] Kuiper C Vissers MCM. Ascorbate as a Co-Factor for Fe- and 2-oxoglutarate dependent dioxygenases: physiological activity in tumor growth and progression. Front Oncol [Internet]. 2014;4:359. 25540771 10.3389/fonc.2014.00359 PMC4261134

[30] Berchner-Pfannschmidt U Yamac H Trinidad B. Nitric oxide modulates oxygen sensing by hypoxia-inducible factor 1-dependent induction of prolyl hydroxylase 2. J Biol Chem. 2007;282:1788–96. 17060326 10.1074/jbc.M607065200

[31] Farah CS. Molecular landscape of head and neck cancer and implications for therapy. Ann Transl Med. 2021;9:915–915. 34164549 10.21037/atm-20-6264 PMC8184465

[32] Ellinghaus P Heisler I Unterschemmann K. BAY 87-2243, a highly potent and selective inhibitor of hypoxia-induced gene activation has antitumor activities by inhibition of mitochondrial complex I. Cancer Med. 2013;2:611–24. 24403227 10.1002/cam4.112 PMC3892793

[33] Koh MY Spivak-Kroizman T Venturini S. Molecular mechanisms for the activity of PX-478, an antitumor inhibitor of the hypoxia-inducible factor-1α. Mol Cancer Ther. 2008;7:90–100. 18202012 10.1158/1535-7163.MCT-07-0463

[34] Inaba K Masui S Iida H. Crystal structures of human Ero1α reveal the mechanisms of regulated and targeted oxidation of PDI. EMBO J. 2010;29:3330–43. 20834232 10.1038/emboj.2010.222 PMC2957217

[35] Tu BP Weissman JS. Oxidative protein folding in eukaryotes. J Cell Biol. 2004;164:341–6. 14757749 10.1083/jcb.200311055 PMC2172237

[36] Nisimoto Y Jackson HM Ogawa H. Constitutive NADPH-dependent electron transferase activity of the Nox4 dehydrogenase domain. Biochemistry. 2010;49:2433–42. 20163138 10.1021/bi9022285 PMC2839512

[37] Maranchie JK Zhan Y. Nox4 is critical for hypoxia-inducible factor 2-α transcriptional activity in von Hippel-Lindau–deficient renal cell carcinoma. Cancer Res. 2005;65:9190–3. 16230378 10.1158/0008-5472.CAN-05-2105 PMC1459967

[38] Drapier JC Hibbs JB. [3] Aconitases: a class of metalloproteins highly sensitive to nitric oxide synthesis. Methods in Enzymology [Internet]. 1996;269:26–36. Elsevier. Available at: https://linkinghub.elsevier.com/retrieve/pii/S0076687996690065. 8791634 10.1016/s0076-6879(96)69006-5

[39] Stiehl DP Wirthner R Köditz J. Increased prolyl 4-hydroxylase domain proteins compensate for decreased oxygen levels. J Biol Chem. 2006;281:23482–91. 16790428 10.1074/jbc.M601719200

[40] Fujita N Markova D Anderson DG. Expression of prolyl hydroxylases (PHDs) is selectively controlled by HIF-1 and HIF-2 proteins in nucleus pulposus cells of the intervertebral disc. J Biol Chem. 2012;287:16975–86. 22451659 10.1074/jbc.M111.334466 PMC3351286

[41] Manuelli V Pecorari C Filomeni G. Regulation of redox signaling in HIF-1-dependent tumor angiogenesis. FEBS J. 2022;289:5413–25. 34228878 10.1111/febs.16110

[42] Yadav S Kalra N Ganju L. Activator protein-1 (AP-1): a bridge between life and death in lung epithelial (A549) cells under hypoxia. Mol Cell Biochem. 2017;436:99–110. 28589371 10.1007/s11010-017-3082-1

[43] Kulisz A Chen N Chandel NS. Mitochondrial ROS initiate phosphorylation of p38 MAP kinase during hypoxia in cardiomyocytes. Am J Physiol Lung Cell Mol Physiol. 2002;282:L1324–9. 12003789 10.1152/ajplung.00326.2001

[44] Singh M Yadav S Kumar M. The MAPK-activator protein-1 signaling regulates changes in lung tissue of rat exposed to hypobaric hypoxia. J Cell Physiol. 2018;233:6851–65. 29665093 10.1002/jcp.26556

[45] Chen Y Chen X Ding X. Afatinib, an EGFR inhibitor, decreases EMT and tumorigenesis of Huh-7 cells by regulating the ERK-VEGF/MMP9 signaling pathway. Mol Med Report [Internet]. 2019;20:3317–25. Available at: http://www.spandidos-publications.com/10.3892/mmr.2019.10562. 10.3892/mmr.2019.10562 PMC6755195 31432165

[46] Mottet D Michel G Renard P. ERK and calcium in activation of HIF-1. Ann N Y Acad Sci. 2002;973:448–53. 12485909 10.1111/j.1749-6632.2002.tb04681.x

[47] Papavassiliou AG Musti AM. The multifaceted output of c-Jun biological activity: focus at the junction of CD8 T cell activation and exhaustion. Cells. 2020;9:24702470. 10.3390/cells9112470 PMC7697663 33202877

[48] Liu C Shi Y Du Y. Dual-specificity phosphatase DUSP1 protects overactivation of hypoxia-inducible factor 1 through inactivating ERK MAPK. Exp Cell Res. 2005;309:410–8. 16081065 10.1016/j.yexcr.2005.06.022

[49] Li Q Ke Q Costa M. Alterations of histone modifications by cobalt compounds. Carcinogenesis. 2009;30:1243–51. 19376846 10.1093/carcin/bgp088 PMC2704281

[50] Lamadema N Burr S Brewer AC. Dynamic regulation of epigenetic demethylation by oxygen availability and cellular redox. Free Radic Biol Med. 2019;131:282–98. 30572012 10.1016/j.freeradbiomed.2018.12.009

[51] Patterson KI Brummer T O’brien PM. Dual-specificity phosphatases: critical regulators with diverse cellular targets. Biochem J. 2009;418:475–89. 19228121 10.1042/bj20082234

[52] Mills CN Joshi SS Niles RM. Expression and function of hypoxia inducible factor-1 alpha in human melanoma under non-hypoxic conditions. Mol Cancer. 2009;8:104. 19919690 10.1186/1476-4598-8-104 PMC2781803

[53] Secades P de Santa-María IS Merlo A. In vitro study of normoxic epidermal growth factor receptor-induced hypoxia-inducible factor-1-alpha, vascular endothelial growth factor, and BNIP3 expression in head and neck squamous cell carcinoma cell lines: Implications for anti-epidermal growth fact: constitutive EGFR-HIF-1α pathway and sensitivity to gefitinib. Head Neck. 2015;37:1150–62. 24798801 10.1002/hed.23733

[54] Rasmussen KD Helin K. Role of TET enzymes in DNA methylation, development, and cancer. Genes Dev. 2016;30:733–50. 27036965 10.1101/gad.276568.115 PMC4826392

[55] Bruine de BL Wachters JE Schrijvers ML. PTEN is associated with worse local control in early stage supraglottic laryngeal cancer treated with radiotherapy. Laryngoscope Investig Otolaryngol. 2019;4:399–404. 10.1002/lio2.272 PMC6703112 31453348

[56] Choudhry H Harris AL. Advances in hypoxia-inducible factor biology. Cell Metab. 2018;27:281–98. 29129785 10.1016/j.cmet.2017.10.005

[57] Masoud GN Li W. HIF-1α pathway: role, regulation and intervention for cancer therapy. Acta Pharmaceutica Sinica B. 2015;5:378–89. 26579469 10.1016/j.apsb.2015.05.007 PMC4629436

[58] Reichmann D Voth W Jakob U. Maintaining a healthy proteome during oxidative stress. Mol Cell. 2018;69:203–13. 29351842 10.1016/j.molcel.2017.12.021 PMC5777170

[59] Ravenna L Principessa L Verdina A. Distinct phenotypes of human prostate cancer cells associate with different adaptation to hypoxia and pro-inflammatory gene expression. Nie D, editor. PLoS One. 2014;9:e96250. 24801981 10.1371/journal.pone.0096250 PMC4011733

[60] Bonello S Zähringer C BelAiba RS. Reactive oxygen species activate the HIF-1α promoter via a functional NFκB site. ATVB. 2007;27:755–61. 10.1161/01.ATV.0000258979.92828.bc 17272744

[61] Betzler AC Theodoraki MN Schuler PJ. NF-κB and its role in checkpoint control. IJMS. 2020;21:3949. 32486375 10.3390/ijms21113949 PMC7312739

[62] Nam SY Ko YS Jung J. A hypoxia-dependent upregulation of hypoxia-inducible factor-1 by nuclear factor-κB promotes gastric tumour growth and angiogenesis. Br J Cancer. 2011;104:166–74. 21119667 10.1038/sj.bjc.6606020 PMC3039796

[63] Wang Y Zhao W Gao Q. pVHL mediates K63-linked ubiquitination of IKKβ, leading to IKKβ inactivation. Cancer Lett. 2016;383:1–8. 27693634 10.1016/j.canlet.2016.09.009

[64] Jiang Y Zhu Y Wang X. Temporal regulation of HIF-1 and NF-κB in hypoxic hepatocarcinoma cells. Oncotarget. 2015;6:9409–19. 25823824 10.18632/oncotarget.3352 PMC4496226

[65] Xu L Pathak PS Fukumura D. Hypoxia-induced activation of p38 mitogen-activated protein kinase and phosphatidylinositol 3′-kinase signaling pathways contributes to expression of interleukin 8 in human ovarian carcinoma cells. Clin Cancer Res. 2004;10:701–7. 14760093 10.1158/1078-0432.ccr-0953-03

[66] Jin P Shin SH Chun YS. Astrocyte-derived CCL20 reinforces HIF-1-mediated hypoxic responses in glioblastoma by stimulating the CCR6-NF-κB signaling pathway. Oncogene. 2018;37:3070–87. 29535421 10.1038/s41388-018-0182-7

[67] Azoitei N Becher A Steinestel K. PKM2 promotes tumor angiogenesis by regulating HIF-1α through NF-κB activation. Mol Cancer. 2016;15:3. 26739387 10.1186/s12943-015-0490-2 PMC4704385

[68] Bommi PV Chand V Mukhopadhyay NK. NER-factor DDB2 regulates HIF1α and hypoxia-response genes in HNSCC. Oncogene. 2020;39:1784–96. 31740787 10.1038/s41388-019-1105-y PMC11095046

